# Random or Stochastic Monoallelic Expressed Genes Are Enriched for Neurodevelopmental Disorder Candidate Genes

**DOI:** 10.1371/journal.pone.0085093

**Published:** 2013-12-27

**Authors:** Aaron R. Jeffries, David A. Collier, Evangelos Vassos, Sarah Curran, Caroline M. Ogilvie, Jack Price

**Affiliations:** 1 Department of Neuroscience, Centre for the Cellular Basis of Behaviour, Institute of Psychiatry, King’s College London, London, United Kingdom; 2 Discovery Neuroscience Research, Eli Lilly and Company Limited, Surrey, United Kingdom; 3 Social, Genetic and Developmental Psychiatry Centre, Institute of Psychiatry, King’s College London, London, United Kingdom; 4 Cytogenetics Department, Guy's and St Thomas' NHS Foundation Trust, London, United Kingdom; Emory University School Of Medicine, United States of America

## Abstract

Random or stochastic monoallelic expressed genes (StMA genes) represent a unique form of monoallelic expression where allelic choice is made at random early in development. The consequential clonal diversity provides opportunity for functional heterozygosity in tissues such as the brain, and can impact on both development and disease. We investigate the relationship of StMA expressed genes previously identified in clonal neural stem cells with the neurodevelopmental disorders autism and schizophrenia. We found that StMA genes show an overrepresentation of schizophrenia risk candidates identified by genome wide association studies from the genetic association database. Similar suggestive enrichment was also found for genes from the NHGRI genome-wide association study catalog and a psychiatric genetics consortium schizophrenia dataset although these latter more robust gene lists did not achieve statistical significance. We also examined multiple sources of copy number variation (CNV) datasets from autism and schizophrenia cohorts. After taking into account total gene numbers and CNV size, both autism and schizophrenia associated CNVs appeared to show an enrichment of StMA genes relative to the control CNV datasets. Since the StMA genes were originally identified in neural stem cells, bias due to the neural transcriptome is possible. To address this, we randomly sampled neural stem cell expressed genes and repeated the tests. After a significant number of iterations, neural stem cell expressed genes did not show an overrepresentation in autism or schizophrenia CNV datasets. Therefore, irrespective of the neural derived transcriptome, StMA genes originally identified in neural stem cells show an overrepresentation in CNVs associated with autism and schizophrenia. If this association is functional, then the regulation (or dysregulation) of this form of allelic expression status within tissues such as the brain may be a contributory risk factor for neurodevelopmental disorders and may also influence disease discordance sometimes observed in monozygotic twins.

## Introduction

Several forms of monoallelic gene expression have been identified. Imprinting, a form of monoallelic gene expression based on parent-of-origin effects, is highly represented in the brain transcriptome [1], and several neurodevelopmental disorders are associated with the disruption of imprinted genes. Genetic cis-acting variants can also influence allelic expression [2] and drive phenotypic diversity. Monoallelic expression of specific disease candidate genes has also been observed in a small number of autism patient samples [3,4]. Another form of monoallelic gene expression, identified by global allelic expression profiling of clonal cell lines, is random or stochastic monoallelic choice (StMA), where the expressed allele within a cell is selected at random early in development and maintained in subsequent cellular progeny [5,6]. This epigenetic driven allelic expression choice contributes to clonal diversity and functional heterozygosity at the cellular level [7], and we have hypothesized that it might, for example, contribute to discordance between monozygotic twins [5]. While the exact impact stochastic monoallelic expression has on development is unclear, our study of allelic expression in human neural stem cells identified a number of neurodevelopmental genes showing this form of allelic expression control [5]. We therefore asked whether stochastic monoallelic expressed genes have any potential significance as a risk factor in the neurodevelopmental disorders, autism and schizophrenia.

## Materials and Methods

We utilized allelic expression measures from a previous study of clonal neural stem cells to construct a list of stochastic monoallelic (StMA) expressed genes [5]. A clone showing monoallelic gene expression must show greater than 2.33 fold difference between the expression levels of the two alleles. StMA gene expression was defined by examining genetically identical sister clonal lines where additional clones showed either biallelic (equal expression of both alleles) or monoallelic expression of the other allele. Since three different neural stem cell sources of different genetic background used (cerebral cortex, striatum and spinal cord), we merged each derived list of StMA genes to form a global gene list used in the enrichment analysis. Similarly, a biallelic gene list was also constructed based on all three genetically identical clonal lines showing biallelic expression. The biallelic lists for each neural stem cell source were then merged together to form a global list for enrichment analysis.

Enrichment analysis was carried out on both association studies (using StMA and biallelic gene lists) and CNV datasets (using StMA gene lists only). Candidate genes from association studies were taken from the Genetic Association Database (http://geneticassociationdb.nih.gov), NHGRI GWAS catalog (http://www.genome.gov/gwastudies) and 81 associated SNPs from the psychiatric genetics consortium [8], where at least one SNP in the LD region reached *P* < 2 × 10^−5^ . Genes mapping close to or containing the risk SNPs in the latter study were identified with Galaxy analysis server [9]. Association study enrichment analysis was carried out by counting the occurrence of StMA and biallelic genes in relation to their expected number of hits (based on the ratio of StMA genes and BA genes). Chi Squared test with Yates’ continuity correction (or Fisher’s Exact test when warned about incorrect approximations) was applied through R using a 2x2 contingency table based on counts vs those StMA and biallelic genes which were not present. 

Disease associated CNV locations were obtained from a number of sources: 75 autism patients from the Brain Body and Genetic Resource Exchange (BBGRE) CNV database (http://bbgre-dev.iop.kcl.ac.uk), 120 autism patients with de novo CNVs from the DECIPHER consortium repository (http://decipher.sanger.ac.uk), 843 autism spectrum disorder patients from a developmental disability CNV study [10], and five CNV regions implicated in large scale autism case-control studies [11]. We also obtained schizophrenia associated recurrent microdeletion CNVs [12] (57 autosomal de novo CNVs from a reduced fecundity population study and a subset of 8 CNVs which also showed co-occurrence in schizophrenia individuals) and also 12 replicated CNVs documented in a recent schizophrenia meta-analysis review [13]. Control CNV datasets from high density oligonucleotide CGH platforms were obtained from the Genome Structural Variation Consortium discovery dataset (validated CNVs), Hapmap individuals [14] and other control datasets with greater than 1,000 mapped variants accessioned in the dbVAR repository (http://www.ncbi.nlm.nih.gov/dbvar ) [14–17]. For enrichment testing, all the control loci from these datasets were combined and referred to as dbVAR controls (individual datasets shown in table S1). Overlapping CNV coordinates within a dataset were merged to define a potential maximally affected region and any RefSeq genes within these CNV regions defined. Since a large number of very short CNVs were in the control CNV datasets, a size selection was applied to only select CNVs greater than 100kb. Disease associated CNVs showed a larger size distribution. CNV coordinates were uploaded to Galaxy and merged to account for any overlapping CNV coordinates. Minimal or critical overlaps of any CNV regions were also identified and a separate analysis carried out (table S2). RefSeq defined genes which overlapped with CNV regions were identified using the join function of Galaxy and accession numbers translated into gene symbols. Published CNV datasets are supplied in File S1. Access to BBGRE and DECIPHER CNV data can be obtained by contacting the database curators. After addition of file headers, the resulting gene/CNV files were then imported into R and enrichment analysis carried out (code supplied, see File S2). In an attempt to minimise size bias issues, we carried out StMA enrichment analysis in three ways – StMA occurrence per 1000 CNV mapped genes, StMA occurrence per megabase of CNV regions and percentage of CNV regions containing StMA genes. A permutation test was also carried out in R to ensure any observed enrichments were not simply a bias from the neural derived transcriptome. Instead of StMA gene counts, we replaced StMA genes with a random sample of neural stem cell expressed genes originally measured in a global allelic expression assay [5]. 10,000 iterations were carried out and enrichment tests performed on each permutation as well as the overall median values obtained.

## Results

### Stochastic Monoallelic Gene Enrichment in Schizophrenia Genome-Wide Association Studies

We used publically available neuropsychiatric datasets to look for enrichment of StMA genes relative to control data. First, occurrence of StMA genes and BA genes within datasets from the genetic association database [18] were examined. Significant enrichments of StMA genes were observed in psychiatric candidate class genes (2.41 fold enrichment, Chi Squared test p-value=0.0007), with a high representation for schizophrenia candidate genes (3.47 fold enrichment, Fishers exact test p-value=0.003). BA genes showed no enrichments in these datasets. No StMA gene enrichments were observed for height (0.36 fold) or diabetes type II (1 fold), both of which like schizophrenia have high heritability from multiple genes of small effect size. Since these datasets probably contain a number of spurious associative results, we investigated more robust genome wide association studies (GWAS) data sources. The manually curated NHGRI GWAS catalog for schizophrenia showed a three-fold enrichment (2 observed, 0.6 expected) of StMA genes. The top schizophrenia hits from the psychiatric genetic consortium genome wide association study [8] showed a 1.95 fold enrichment (3 observed, 1.54 expected). While enriched, neither showed statistical significance possibly due to the low number of available genes. 

### Stochastic Monoallelic Gene Enrichment in Autism and Schizophrenia associated CNVs

**Figure 1:**
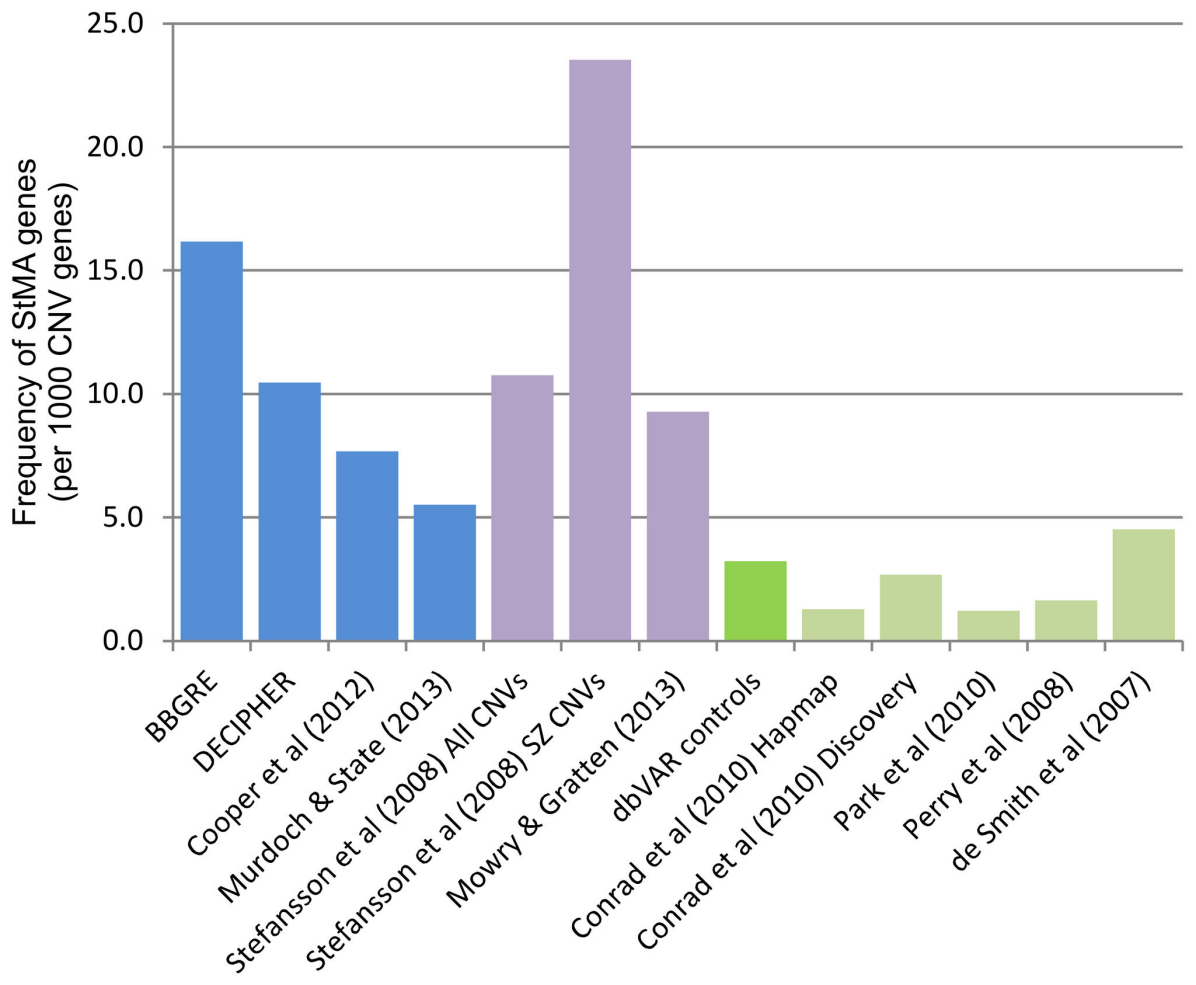
Representation of StMA gene occurrence (per 1000 CNV mapped genes) in autism CNV datasets (blue), schizophrenia (purple) and control datasets (green). The “dbVAR controls” represents a reference dataset created from the merger of all CNV loci derived from a series of control population datasets (shown in light green).

Since allelic status of StMA genes correlates with overall transcript levels [5,6,19], such genes have the potential to be dosage sensitive. This led us to consider whether an enrichment of StMA genes exists in copy number variants (CNVs) associated with neurodevelopmental disorders. We therefore investigated a series of autism and schizophrenia CNVs in relation to non-pathogenic CNVs as defined in population control survey datasets obtained from the database of genomic structural variation (dbVAR) [14–16,20]. Genes were mapped to the regions covered by the CNVs and the frequency of StMA genes examined in relation to total number of CNV genes, CNV region number and size (Table 1 and Figure 1). Analysis was also performed on disease based minimal CNV regions, where overlapping CNVs used to define a minimal critical region. Similar results were obtained (table S1).

**Table 1:** Summary of CNV, gene content and StMA occurrence in autism, schizophrenia and reference control datasets.

	***Autism***				***Schizophrenia***			***Reference***
	**BBGRE**	**DECIPHER**	**Cooper et al (2012)**	**Murdoch & State (2013)**	**Stefansson et al (2008**)** All CNVs**	**Stefansson et al (2008**)** SZ CNVs**	**Mowry & Gratten (2013)**	**dbVAR controls**
Total Genes Mapped to CNV regions	866	5256	5864	181	929	85	323	2781
StMA Genes	14	55	45	1	10	2	3	9
*Permutation StMA Genes*	*6*	*44*	*49*	*1*	*8*	*0*	*1*	*18*
StMA/1000 CNV Genes	16.2	10.5	7.7	5.5	10.8	23.5	9.3	3.2
*Permutation Genes/1000 CNV genes*	*6.9*	*8.4*	*8.4*	*5.5*	*8.6*	*0*	*3.1*	*6.5*
Enrichment relative to dbVAR controls	5	3.23	2.37	1.71	3.33	7.27	2.87	1
*Permutation enrichment relative to dbVAR controls*	*1.15*	*1.31*	*1.31*	*0.71*	*1.33*	*0*	*0.78*	*1*
StMA Enrichment Test p-value	0.000078	0.000849	0.02143	0.4682	0.01297	0.0402	0.1211	-
*Permutation Test p-value (median)*	*1*	*0.4286*	*0.4228*	*1*	*0.6531*	*1*	*0.7132*	*-*
CNV region total size (Mb)	84.15	644.25	504.87	8.41	94.29	5.99	21.3	215.18
CNV region mean size (Mb)	1.22	3.25	0.78	1.68	1.65	0.75	1.78	0.49
Number of CNV regions	69	198	650	5	57	8	12	440
CNVs containing StMA	13 (18.8%)	40 (20.2%)	39 (6.0%)	1 (20%)	8 (14.0%)	2 (25%)	3 (25%)	9 (2.0%)
StMA CNV Enrichment Test p-value	3.33E-07	5.84E-15	0.003	0.1079	0.0002	0.0148	0.0028	-
Genes/Mb CNV region	10.3	8.2	11.6	21.5	9.9	14.2	15.2	12.9
StMA Genes/Mb CNV region	0.166	0.085	0.089	0.119	0.106	0.334	0.141	0.042
*Permutation StMA Genes/Mb CNV region*	0.071	0.068	0.097	0.119	0.085	0	0.047	0.084
Enrichment relative to dbVAR controls	4	2	2.1	2.8	2.5	8	3.4	1
*Permutation Enrichment relative to dbVAR controls*	0.9	0.8	1.2	1.4	1	0	0.6	1

Shaded rows indicate equivalent results where random genes sampled from the expressed neural stem cell transcriptome were used (based on median values after 10,000 iterations). Proportion of StMA genes relative to total number of genes is shown, together with the gene and StMA density of for each CNV dataset. Each disease dataset was tested against ‘dbVAR controls’, a merger of CNV locations from series of control/population based CNV studies (see table S2). Enrichment significance relative to the dbVAR control is shown, with a Chi Squared test p-values (or Fisher’s exact test when gene counts were low) applied to the StMA gene counts relative to the total number of genes and also number of CNVs with StMA genes.

Disease datasets were obtained from CNV databases and publications. Autism CNVs from BBGRE and DECIPHER databases show high enrichment of StMA gene occurrence relative to the total number of genes (3.2-5.0 fold). The number of CNV regions containing StMA genes also showed a higher proportion of autism and schizophrenia CNV regions containing StMA genes (14 to 25%) compared with dbVAR control CNVs (2%). When the CNV size is taken into account to calculate gene density, the frequency of StMA genes per megabase of CNV was 2 to 4 fold higher than the dbVAR control CNV regions. The remaining two autism publication derived datasets represent a comprehensive CNV morbidity map of neurodevelopmental delay, with some of the patients having an autism spectrum disorder diagnosis [10] and 5 genomic regions which are recurrent de novo CNV hotspots for autism [11]. The first study shows StMA with lower but still statistically significant overrepresentation of StMA genes. However, counts of the mapped gene occurring in the five autism genomic hotspot regions showed enrichments but failed to show statistical significance.

The schizophrenia datasets were obtained from two publications. We used 12 replicated schizophrenia CNVs documented in a recent meta-analysis review by Mowry and Gratten (2013) [13] and also reduced fecundity rare de novo CNVs identified by Stefansson et al (2008) [12], a subset of which showed occurrence in schizophrenia cohorts (labelled as SZ). The 12 replicated CNV loci showed StMA gene overrepresentation based on direct gene counts although statistical significance was not achieved. The study by Stefansson et al showed StMA enrichment together with statistical significance for both the 57 autosomal rare de novo CNVs and also the subset of these which showed co-occurrence in schizophrenia cohorts.

### Testing for Neural Stem Cell Transcriptome Enrichment in Autism and Schizophrenia CNVs

Since our identified StMA genes were originally identified in neural stem cells [5], it is conceivable that the enrichments described with autism and schizophrenia CNVs are from the neural derived transcriptome rather than the StMA genes. To test this, we replaced the StMA gene list with randomly sampled neural stem cell expressed genes which were originally assayed in the allelic expression assay. Each gene list permutation was tested against the disease CNV vs the dbVAR control CNV gene list. After 10,000 iterations, no enrichment was found at the gene count level (based on median gene counts or Bonferroni adjusted p-values of Fisher’s exact test applied at each iteration) for autism or schizophrenia associated CNVs when compared to dbVAR control CNVs. StMA genes are therefore overrepresented in autism and schizophrenia associated CNVs rather than a biased observation originating from the neural stem cell transcriptome.

## Discussion

We have demonstrated an associative link of neural stem cell identified StMA genes with autism and schizophrenia GWAS and CNV identified genes. Pathological neuropsychiatric-associated CNVs are likely to alter the transcript levels of specific risk genes [21]. Similarly, variants associated with schizophrenia risk are enriched for expression quantitative trait loci [22]. The precise role of StMA genes is still unclear, although monoallelic expression in clonal cells show lower overall transcript levels compared to clones expressing the gene biallelically [5,6,19]. The random allelic choice of StMA genes in the cell population of a given tissue may therefore represent a form of gene dosage control. The additional influence of CNV deletions, duplications or genetic regulatory variants can directly impact on the allelic expression composition of the cell population and alter the gene dosage in a tissue [23], which for some genes will contribute to increased risk of disease penetrance. The epigenetic profile at CNVs is also likely to have a bearing on gene dosage and phenotype. Repressive epigenetic marks such as DNA methylation usually associate with the silenced allele at monoallelic expressed genes. In the case of a deletion, presence or absence of repressive epigenetic marks on the remaining allele will dictate whether the cell will express any transcript from that gene. 

Consistent with our disease enrichment findings, allelic imbalances identified within induced pluripotent stem cell derived differentiated human neurons also show a modest association for schizophrenia and autism candidate genes [24]. Allele specific histone modifications in hESCs have also been associated with genes linked to developmental syndromes mediated by deletions [25]. How then could StMA genes contribute to neurodevelopmental disorders in the absence of a CNV? Genes undergoing such allelic control select which allele to express early in development. The resulting mosaicism of allelic expression at the cellular level would generate clonal functional heterozygosity [7]. Since cell lineage studies suggest that clones of cells disperse widely through the cerebral cortex during fetal development [26], StMA expression would be predicted to drive cellular diversity widely as neurogenesis progresses. Phenotypic diversity could arise because of polymorphisms/mutations in one or both alleles, or through reduced gene dosage associated with monoallelic expressing cells [5,6,19]. Either scenario can lead to the selective expression of risk alleles leading to increased risk of disease penetrance. This may be further reinforced through bias in the choice of one allele over another, an effect observed in a subset of StMA genes (our unpublished observations and Zwemer et al (2012) [27]). Such clonal distributions could also be a contributory factor to disease discordance observed in monozygotic twins. StMA gene identification may also represent a unique way to identify novel putative disease risk candidate genes.

In summary, we have previously identified genes showing stochastic monoallelic expression patterns in clonal neural stem cells, together with a number of genetic and epigenomic associations [5]. Here we demonstrate that these same genes are overrepresented in candidate risk genes for autism and schizophrenia. This is consistent with a similar disease association for allelic imbalances observed in differentiated human neurons [24] and allele specific histone modifications in human embryonic stem cells [25]. Stochastic allelic choice may therefore be a contributory factor in disease etiology and warrants further investigation.

## Supporting Information

Table S1 **Control CNV datasets used to construct the dbVAR control dataset.** Total gene content and StMA gene occurrence are shown for each data set. The Conrad et al (2010) control study consisted of two CNV lists – a CNV discovery dataset and HapMap based dataset as shown in the table.
(DOCX)

Table S2 **Summary of CNV data sets based on genes mapped to minimal overlap CNV coordinates.** Total gene content and StMA gene occurrence are shown for autism (BBGRE and DECIPHER) and schizophrenia (Cooper et al 2012 and Stefansson et al 2008) CNV datasets based on genes mapped to minimal overlap CNV coordinates. Enrichment tests were carried out relative to dbVAR controls, a merger of CNV loci as shown in table S2.
(DOCX)

File S1 **Allelic expression gene lists and CNV coordinates and their associated genes used in the study.**
(XLSX)

File S2 **R code used for CNV enrichment analysis and permutation test.**
(TXT)
