# Supplementary material for: Random or Stochastic Monoallelic Expressed Genes Are Enriched for Neurodevelopmental Disorder Candidate Genes
Source: PLoS One. 2013 Dec 27;8(12):e85093. doi: 10.1371/journal.pone.0085093 (PMC3874034; doi:10.1371/journal.pone.0085093)
Supplement: Table S1 — Control CNV datasets used to construct the dbVAR control dataset. Total gene content and StMA gene occurrence are shown for each data set. The Conrad et al (2010) control study consisted of two CNV lists – a CNV discovery dataset and HapMap based dataset as shown in the table. (DOCX) [file pone.0085093.s001.docx]

Table S1. Control CNV datasets used to construct the dbVAR control dataset.

|  | **Conrad et al (2010) Hapmap** | **Conrad et al (2010) Discovery** | **Park et al (2010)** | **Perry et al (2008)** | **de Smith et al (2007)** |
| --- | --- | --- | --- | --- | --- |
| Total Genes Mapped to CNV regions | 781 | 373 | 815 | 1212 | 883 |
| StMA Genes | 1 | 1 | 1 | 2 | 4 |
| StMA/1000 CNV Genes | 1.3 | 2.7 | 1.2 | 1.7 | 4.5 |
|  |  |  |  |  |  |
| CNV region total size (Mb) | 50.83 | 25.66 | 35.45 | 75.61 | 69.93 |
| CNV region mean size (Mb) | 0.28 | 0.29 | 0.22 | 0.41 | 0.55 |
| Number of CNV regions | 182 | 89 | 161 | 184 | 128 |
| CNVs containing StMA | 1 (0.5%) | 1 (1.1%) | 1 (0.6%) | 2 (1.1%) | 4 (3.1%) |
|  |  |  |  |  |  |
| Genes/Mb CNV region | 15.4 | 14.5 | 23.0 | 16.0 | 12.6 |
| StMA Genes/Mb CNV region | 0.020 | 0.039 | 0.028 | 0.026 | 0.057 |

Total gene content and StMA gene occurrence are shown for each data set. The Conrad et al (2010) control study consisted of two CNV lists – a CNV discovery dataset and HapMap based dataset as shown in the table.
