# Supplementary material for: Random or Stochastic Monoallelic Expressed Genes Are Enriched for Neurodevelopmental Disorder Candidate Genes
Source: PLoS One. 2013 Dec 27;8(12):e85093. doi: 10.1371/journal.pone.0085093 (PMC3874034; doi:10.1371/journal.pone.0085093)
Supplement: Table S2 — Summary of CNV data sets based on genes mapped to minimal overlap CNV coordinates. Total gene content and StMA gene occurrence are shown for autism (BBGRE and DECIPHER) and schizophrenia (Cooper et al 2012 and Stefansson et al 2008) CNV datasets based on genes mapped to minimal overlap CNV coordinates. Enrichment tests were carried out relative to dbVAR controls, a merger of CNV loci as shown in table S2. (DOCX) [file pone.0085093.s002.docx]

Table S2. Summary of CNV data sets based on genes mapped to minimal overlap CNV coordinates.

|  | **BBGRE** | **DECIPHER** | **Cooper et al (2012)** | **Stefansson et al (2008)** | **dbVAR controls** |
| --- | --- | --- | --- | --- | --- |
| Total Genes Mapped to CNV regions | 850 | 2849 | 2335 | 775 | 2781 |
| StMA Genes | 14 | 27 | 21 | 8 | 9 |
| StMA/1000 Genes | 16.47058824 | 9.477009477 | 8.993576017 | 10.32258065 | 3.236245955 |
| Enrichment relative to dbVAR controls | 5.1 | 2.9 | 2.8 | 3.2 | 1.0 |
| StMA Enrichment Test p-value | 0.000061 | 0.005607 | 0.012330 | 0.007592 |  |
|  |  |  |  |  |  |
| CNV region total size (Mb) | 82.47 | 312.83 | 196.91 | 81.76 | 215.18 |
| CNV region mean size (Mb) | 1.20 | 1.48 | 0.28 | 1.41 | 0.49 |
| Number of CNV regions | 69 | 211 | 691 | 58 | 440 |
| CNV regions containing StMA | 14 (20.3%) | 27 (12.8%) | 21 (3.0%) | 8 (13.8%) | 9 (2.0%) |
| StMA CNV Enrichment Test p-value | 6.25E-08 | 5.52E-08 | 0.4099 | 0.0002 |  |
|  |  |  |  |  |  |
| Genes/Mb CNV region | 10.3 | 9.1 | 11.9 | 9.5 | 12.9 |
| StMA Genes/Mb CNV region | 0.170 | 0.086 | 0.107 | 0.098 | 0.042 |
| Enrichment relative to dbVAR controls | 4.1 | 2.1 | 2.5 | 2.3 | 1.0 |

Total gene content and StMA gene occurrence are shown for autism (BBGRE and DECIPHER) and schizophrenia (Cooper et al 2012 and Stefansson et al 2008) CNV datasets based on genes mapped to minimal overlap CNV coordinates. Enrichment tests were carried out relative to dbVAR controls, a merger of CNV loci as shown in supporting information table S2.
